# Evaluation of physical and chemical isolation methods to extract and purify *Campylobacter jejuni* extracellular polymeric substances

**DOI:** 10.3389/fmicb.2024.1488114

**Published:** 2024-10-25

**Authors:** Natalija Pavlinjek, Anja Klančnik, Jerica Sabotič

**Affiliations:** ^1^Department of Food Science and Technology, Biotechnical Faculty, University of Ljubljana, Ljubljana, Slovenia; ^2^Department of Biotechnology, Jožef Stefan Institute, Ljubljana, Slovenia

**Keywords:** *Campylobacter jejuni*, biofilm, extracellular polymeric substances, isolation methods, extraction and purification

## Abstract

The pathogenic bacterium *Campylobacter jejuni* is a major food safety concern as it can form biofilms that increase its survival and infective potential. Biofilms consist of microbial cells and extracellular matrix (ECM), which is made of water and extracellular polymeric substances (EPS), which are critical for structural integrity and pathogenicity. The aim of this study was to optimize a protocol for the isolation of *C. jejuni* ECM. We employed eight physical and chemical isolation methods to extract and purify ECM, followed by different qualitative and quantitative analyses using gel electrophoresis and spectroscopy. This comprehensive approach enabled the evaluation of ECM composition in terms of polysaccharides, proteins, and extracellular DNA. The isolation methods resulted in different yields and purities of the extracted ECM components. Centrifugation in combination with chemical treatments proved to be most effective, isolating higher concentrations of polysaccharides and proteins. Additionally, extraction with ether solution facilitated the recovery of high-molecular-weight extracellular DNA. Overall, we provide a refined methodology for ECM extraction from *C. jejuni*. As polysaccharides and proteins participate in biofilm stability and microbial communication, and extracellular DNA participates in genetic exchange and virulence, our study contributes towards a better understanding of the persistence of this pathogen in the food industry.

## 1 Introduction

The Gram-negative bacterium *Campylobacter jejuni* is found in the intestines of many wild and domestic animals, making them potential asymptomatic carriers or zoonotic transmission (Blaser, 1997; Snelling et al., 2005; Burnham and Hendrixson, 2018). It is one of the main causes of bacterial foodborne gastroenteritis worldwide and the most common cause of foodborne zoonotic infections (EFSA and ECDC, 2023). Infections in humans usually occur through the ingestion of contaminated food of animal origin or untreated water or direct contact with infected animals, particularly pets. However, most human cases are associated with the consumption of contaminated poultry (Blaser, 1997; Sheppard and Maiden, 2015). *C. jejuni* has an optimal growth temperature of around 42°C, which facilitates its colonization in chicken intestine and makes poultry an important vector for its transmission into the human food chain (Snelling et al., 2005; Levin, 2007; Sheppard and Maiden, 2015; Burnham and Hendrixson, 2018).

Contrary to previous assumptions that *C. jejuni* cannot survive outside hosts in aerobic natural environments or in the food chain (Solomon and Hoover, 1999; Klančnik et al., 2013, 2014; Giaouris et al., 2015), it now shows a wide distribution in the environment and has been detected in food, water, and microbial biofilms on microplastics from seawater (Good et al., 2019; Tram et al., 2020; Kolenc et al., 2024). Recent studies have further elucidated its pathogenesis, persistence, and resilience and have linked these properties to genomic polymorphism, limited catabolic capacity, abnormalities in gene regulation, and a protective biofilm matrix that shields it from environmental stressors (Klančnik et al., 2021; Sabotič et al., 2023).

The formation of biofilms is an important survival strategy for *C. jejuni*, providing protection against environmental stress and increasing its infectivity. These biofilms comprise dynamic microbial communities that form on both abiotic and biotic surfaces and are driven by multiple cellular interactions and complex adhesion mechanisms. Biofilms can rapidly (within 48 h) develop into dense structures with strong adhesion and structural complexity and are thus difficult to treat (Sabotič et al., 2023; Joshua et al., 2006; Teh et al., 2010; Reuter et al., 2010; Püning et al., 2021; Ramić et al., 2023; Ma et al., 2022; Carpentier and Cerf, 1993; Sulaeman et al., 2012; Kemper and Hensel, 2023).

Biofilms are complex assemblies of microorganisms embedded in an extracellular matrix (ECM) of water and extracellular polymeric substances (EPS; Aguilera et al., 2008). EPS are crucial for the formation, architecture, and functionality of biofilms and account for 50–90% of biofilm mass. They include polysaccharides, proteins, lipids, and extracellular DNA (eDNA) at different concentrations, depending on environmental conditions and nutrient availability (Donlan, 2002; Vu et al., 2009; Flemming et al., 2016; Karygianni et al., 2020; Flemming et al., 2023).

ECM plays a crucial role in improving the resistance of *C. jejuni* biofilms to environmental stress. It forms a protective barrier around cells that not only protects against physical disturbances, antimicrobial agents, bacteriophages, and biocides but also contributes to the mechanical strength and stability of the biofilm. This barrier increases the antimicrobial resistance of the biofilm by hindering the diffusion of antibiotics and complicating the treatment of associated infections by promoting the intercellular exchange of resistance genes (Donlan, 2002; Karygianni et al., 2020; Flemming et al., 2016). The bacterial ECM also significantly affects the heterogeneity of biofilms by influencing porosity, density, and water content, thereby improving the adaptability of biofilms to different environmental conditions. In addition, bacterial ECM promotes unique microenvironments by enriching biofilms with nutrients and supporting vital functions, such as resource acquisition, hydration, and external digestion, all of which are important for cell survival, metabolic activity, and intercellular interactions. The bacterial ECM contains environmental materials, such as dissolved nutrients, humic substances, and exopolymer particles, which are crucial for cellular metabolism and the structural integrity of the biofilm. Furthermore, most bacterial ECM consist of neutral or polyanionic polysaccharides, which are crucial for the attraction of divalent cations such as calcium and magnesium. These cations bind to bacterial ECM polymers and form hydrogen bonds that stabilize biofilm structure, provide structural cohesion, and protect the biofilm from desiccation (Donlan, 2002). Environmental factors, such as temperature, pH, nutrient availability, and stress, influence bacterial regulatory pathways and lead to increased bacterial ECM production and modifications (Moorhead and Griffiths, 2011; Lu et al., 2012; Feng et al., 2016). Components of ECM, such as eDNA, can be degraded by enzymes or external factors, leading to the dissolution of biofilms and potential bacterial proliferation (Brown et al., 2015; Flemming et al., 2016; Karygianni et al., 2020). This degradation underlines the dynamic nature of biofilms and their susceptibility to environmental influences.

ECM is also central to the formation and maintenance of *C. jejuni* biofilms, thereby increasing biofilm resistance and facilitating *C. jejuni* spread. In *C. jejuni*, ECM plays a complex role in biofilms and are thus important for microbial ecology, pathogenesis, and treatment resistance (Table 1). Different methods have been used to study ECM characteristics, composition and function (Table 2). Crystal violet staining was usually used to quantify biofilms, which is essential for the estimation of ECM content (Feng et al., 2016; Oh et al., 2018). Fluorescence microscopy was used for ECM visualization, with methods such as high-content screening with TAMRA and SytoX fluorescent markers providing quantitative insights into the integrity and composition of ECM (Oh et al., 2018; Whelan et al., 2021).

**Table 1:** The roles of extracellular polymeric substances (EPS) in *Campylobacter jejuni* biofilms. eDNA: extracellular DNA.

Role of EPS	Key findings	Bacteria studied	Study
EPS is crucial for the integrity and protection of biofilms. The EPS matrix mediates cell-to-cell communication and protects microorganisms against environmental stress.	Diallyl sulphide exerts strong antimicrobial activity against sessile *C. jejuni* cells by disrupting the EPS structure of biofilms.	*C. jejuni*	Lu et al. (2012)
EPS provides structural support and protection against aerobic stress.	Dual-species *C. jejuni* biofilms show enhanced survival under aerobic stress, attributed to higher amounts of and more diverse chemical compositions of EPS compared to mono-species biofilms. EPS contributes to the structural integrity, water retention, and resistance to desiccation of biofilms, thereby protecting *C. jejuni*.	*C. jejuni*, *Staphylococcus aureus*, *Salmonella enterica*, and *Pseudomonas aeruginosa*	Feng et al. (2016)
Iron supplementation increased biofilm formation by stimulating the production of eDNA and EPS.	EPS production was stimulated by iron, which contributed to the formation of biofilm matrices encasing *C. jejuni* and possibly helped decrease exposure to oxygen and other stress conditions.	*C. jejuni*	Oh et al. (2018)
EPS support the structural stability and improve substrate exchange and nutrient circulation in biofilms.	Cinnamaldehyde was effective in inhibiting and degrading *Campylobacter* biofilms, influencing auto-aggregation, motility, and EPS production.	*C. jejuni* and *C. coli*	Yu et al. (2020)
Exposure to pancreatic amylase results in secretion of *α*-dextran, a component of biofilm EPS, enhancing biofilm formation.	Exposure to pancreatic amylase results in secretion of α-dextran, a component of biofilm exopolymeric matrix, enhancing biofilm formation.	*C. jejuni*	Jowiya et al. (2015)
EPS provide a protective matrix for biofilms but is penetrable by ZnO nanoparticles.	ZnO nanoparticles penetrate EPS and cause cell death without damaging EPS structure. The inactivation mechanism involves alterations in quinone structures and DNA damage likely due to reactive oxygen species generated by the ZnO nanoparticles.	*C. jejuni*	Lu et al. (2012)
Autoinducer-2 might influence EPS composition, as it affects biofilm density and viability.	Autoinducer-2 affected the expression of virulence genes, which could be related to changes in EPS composition and function.	*C. jejuni*	Moorhead and Griffiths (2011)
eDNA is a major component of *C. jejuni* biofilms. eDNA facilitates the initial attachment, establishment, and maintenance of biofilms and bacterial allocation.	Environmental stress induces bacterial lysis, which leads to the release of eDNA and formation of *C. jejuni* biofilms.	*C. jejuni*	Feng et al. (2018)
eDNA is required for the maturation and three-dimensional development of biofilms. It is not involved in initial adhesion but is released following flagella-mediated bacterial attachment.	DNase treatment degrades eDNA and thereby disrupts biofilms, which highlights the role of eDNA in biofilm integrity and stress tolerance.	*C. jejuni*	Svensson et al. (2014)
eDNA is an essential component of *C. jejuni* biofilms when attached to stainless steel surfaces. It provides hydration, traps nutrients, and reduces access to antimicrobials. Biofilms allow genetic transfer of antibiotic resistance, which might occur through natural transformation facilitated by eDNA within the biofilm.	eDNA is present in *C. jejuni* biofilms under both aerobic and microaerobic conditions and contributes to biofilm formation and structure.	*C. jejuni*	Brown et al. (2015)

**Table 2:** The methods used in studies of *Campylobacter jejuni* extracellular polymeric substances (EPS).

Method category	Techniques	Purpose	Common applications	Study
Extraction and purification	Centrifugation and ethanol precipitation	To isolate EPS and purify it from other biofilm components.	Purification of cells and other biofilm components from biofilm.	Yu et al. (2020)
Extraction and purification	Cold acetone precipitation	To isolate and purify EPS from biofilms.	Isolation of EPS from bacterial cultures.	Jowiya et al. (2015)
Biofilm quantification	Crystal violet staining	To quantify biofilm biomass, indicating the potential quantity of EPS within biofilms.	Standard method for biofilm biomass determination.	Feng et al. (2016), Oh et al. (2018)
EPS analysis	High-content screening with TAMRA and SytoX fluorescent markers	To quantitatively assess the integrity and composition of EPS in adherent *C. jejuni* biofilms under aerobic conditions.	Analysis of homogeneity and consistency of biofilm formation.	Whelan et al. (2021)
EPS analysis	Confocal microscopy and staining	To study the production and effects of cell-signaling compounds on EPS characteristics and biofilm formation.	Determination of the molecular composition of biofilms.	Moorhead and Griffiths (2011)
Component characterization	Raman spectroscopy in combination with confocal laser scanning microscopy	To determine the chemical composition of EPS.	Imaging and molecular analysis of biofilm structure and EPS matrices.	Feng et al. (2016)
Component characterization	Scanning electron microscopy	To visualize biofilm architecture and provide insights into the EPS matrix within the biofilm.	Morphological analysis of biofilms and their EPS matrix.	Melo et al. (2017)
Component characterization	Phenol-sulfuric acid assay	To quantify total carbohydrates in EPS.	Quantification of polysaccharide content in EPS.	Jowiya et al. (2015)
Component characterization	Fourier transform infrared spectroscopy, Raman spectroscopy, scanning electron microscopy	To characterize the biochemical composition and structural integrity of EPS after ZnO nanoparticle treatment and observe interactions between nanoparticles, EPS, and cells in biofilm.	Studying the impact of nanoparticles on biofilms, bacterial cells, and EPS.	Lu et al. (2012)
Component characterization and structure determination	Fluorescence lectin-binding analysis	To characterize glycoconjugates in the EPS matrix.	Visualization and analysis of EPS components in *C. jejuni* biofilms.	Turonova et al. (2016)
Detailed characterization of components	Nuclear magnetic resonance	To characterize the molecular structure of EPS.	Characterization of the molecular structure of EPS.	Jowiya et al. (2015)
Biofilm matrix composition	Fluorescence microscopy	To analyze the presence of eDNA and EPS within the biofilm matrix.	Visualization of biofilm structure and EPS components.	Oh et al. (2018)
Biofilm matrix composition	Stability test with proteinase K and sodium metaperiodate and crystal violet staining	To assess the structural roles of proteins and carbohydrates in the biofilm EPS matrix and to quantify the remaining biofilm biomass after stability tests.	Evaluation of biofilm resistance to enzymatic degradation of EPS. Measurements of biofilm mass and the presence of EPS.	Melo et al. (2017)
Monosaccharide composition analysis	High-performance anion-exchange chromatography	Monosaccharide analysis of EPS.	Glycan composition analysis of EPS.	Jowiya et al. (2015)
Molecular techniques	SDS-PAGE	To analyze the protein profile of EPS components.	Analysis of the protein profile of EPS components.	Yu et al. (2020)
Genetic analysis	Pulsed-field gel electrophoresis	To understand genetic diversity that affects EPS composition and biofilm formation.	Genetic typing of bacterial strains in biofilm studies.	Melo et al. (2017)

The techniques used to characterize ECM components range from microscopic methods such as confocal microscopy and scanning electron microscopy to spectroscopic methods such as Raman spectroscopy and Fourier transform infrared spectroscopy, which help determine the biochemical composition of ECM in detail (Moorhead and Griffiths, 2011; Feng et al., 2016; Melo et al., 2017). In addition, high-performance anion exchange chromatography and nuclear magnetic resonance provide more precise details about the molecular structure of ECM components (Jowiya et al., 2015). Moreover, molecular techniques and genetic analyses link ECM components to their functional roles (Melo et al., 2017; Yu et al., 2020). Quantitative PCR with SYBR Green I and specific primers is used to investigate specific components (e.g., eDNA), also in combination with confocal microscopy (Feng et al., 2018). DNase-I is used to investigate the roles of flagella-mediated adhesion and eDNA in biofilm formation and maturation (Svensson et al., 2014; Feng et al., 2018; Brown et al., 2015). Fluorescence lectin binding analysis is used to characterize glycoconjugates in ECM, providing insights into the complex interactions within biofilms (Turonova et al., 2016).

The aim of this study was to optimize a protocol that isolates the ECM of *C. jejuni* and to evaluate its composition. We investigated different physical and chemical methods for isolating essential ECM components, such as polysaccharides, proteins, and eDNA, to determine the most effective techniques to extract these molecules at high concentrations.

## 2 Materials and methods

### 2.1 Growth conditions

Cultures of *C. jejuni* ATCC 11168 were stored at −80°Cin 20% glycerol (Kemika, Croatia) and 80% Mueller Hinton broth (MHB, Oxoid, UK). *C. jejuni* was incubated on Karmali agar (Oxoid, UK) supplemented with *Campylobacter*-selective Karmali supplement (Oxoid, UK) for 24 h under microaerobic conditions (5% O_2_, 10% CO_2_, and 85% N_2_; Thermo Scientific Oxoid CampyGen atmosphere, USA) in anaerobic jar (3.5 L, Oxoid, UK) in incubator (Kambič, Slovenia) at 42°C. The pure culture was transferred to Mueller Hinton agar (MHA, Oxoid, UK) and incubated under the same conditions overnight.

### 2.2 ECM isolation

Eight different methods were used to isolate the ECM, all of which were preceded by the same step. *C. jejuni* biomass was scraped off four plates using a sterile disposable cotton swab and added to 1.5 ml microcentrifuge tubes containing 1 ml of phosphate-buffered saline (PBS; 10 mM Na_2_HPO_4_, 1.8 mM KH_2_PO_4_, 137 mM NaCl, and 2.7 mM KCl, pH 7.4). Suspension was centrifuged at 12,000 × *g* for 3 min at 4°C. The supernatant with weakly bound ECM components (named as method SV) was then removed using an automated pipette, filtered through a 0.2 μm pore size membrane (Whatman, UK), and stored at −20°C until further use. The obtained biomass pellet was used for the eight isolation methods (named A–H) described below. The isolated ECM was stored at −20°C until further use.

#### 2.2.1 Isolation with weakly bound ECM (named as method SV)

The supernatant containing weakly bound ECM components was obtained after centrifugation of the resuspended *C. jeuni* biomass at 12,000 × *g* for 3 min at 4°C. It was removed using an automated pipette, filtered through a 0.2 μm pore size membrane (Whatman, UK), and stored at −20°C until further use.

#### 2.2.2 Isolation with NaCl (named as method A)

The pellet was resuspended in 1 ml of 1.5 M NaCl (KEFO 7647-14-5) solution by vortexing. This suspension was centrifuged at 5,000 × *g* for 10 min at 25°C, and the supernatant, containing the isolated ECM, was filtered through a 0.2 μm pore size membrane (Whatman, UK; Chiba et al., 2015).

#### 2.2.3 Isolation by centrifugation (named as method B)

The pellet was resuspended in 1 ml of PBS (10 mM Na_2_HPO_4_, 1.8 mM KH_2_PO_4_, 137 mM NaCl, and 2.7 mM KCl, pH 7.4) by vortexing. This suspension was centrifuged at 20,000 × *g* for 20 min at 4°C, and the supernatant, containing the isolated ECM, was filtered through a 0.2 μm pore size membrane (Whatman, UK; Liu and Fang, 2002).

#### 2.2.4 Isolation by heating in Na_2_CO_3_ (named as method C)

The pellet was resuspended in 1 ml of PBS by vortexing, and this suspension was transferred into a new 1.5 ml microcentrifuge tube with 5 mg of Na_2_CO_3_ (Honeywell Fluka 31432). The solution was then incubated for 35 min in a ThermoShaker thermoblock at 80°C with simultaneous stirring at 400 rpm and then cooled at room temperature and centrifuged at 12,000 × *g* for 20 min at 4°C (Felz et al., 2016). The supernatant, containing the isolated ECM, was filtered through a 0.2 μm pore size membrane (Whatman, UK).

#### 2.2.5 Isolation with ethylenediaminetetraacetic acid (EDTA; named as method D)

The pellet was resuspended in 1 ml of PBS by vortexing, and the suspension was divided into two 2 ml microcentrifuge tubes for further steps, to each of which 500 μl of 2% EDTA (Serva 11280.02) was added to give a final EDTA concentration of 1%. This was followed by incubation with agitation on an orbital shaker for 3 h at 4°C and then centrifugation at 12,000 × *g* for 20 min at 4°C (Jachlewski et al., 2015). The supernatant, containing the isolated ECM, was filtered through a 0.2 μm pore size membrane (Whatman, UK).

#### 2.2.6 Isolation with NaOH (named as method E)

The pellet was resuspended in 1 ml of PBS by vortexing, and 0.4 g of NaOH (Fisher 1310-73-2) was added. This was followed by incubation with agitation on an orbital shaker for 3 h at 4°C and centrifugation at 20,000 × *g* for 20 min at 4°C (Jachlewski et al., 2015). The supernatant, containing the isolated ECM, was filtered through a 0.2 μm pore size membrane (Whatman, UK).

#### 2.2.7 Isolation with formaldehyde and NaOH (named as method F)

The pellet was resuspended in 1 ml of PBS by vortexing, and 6 μl of 37% formaldehyde (Sigma-Aldrich 1.04003.1000, Merck, Germany) was added. This was followed by incubation with agitation on an orbital shaker for 1 h at 4°C. Next, 0.4 ml of 1 M NaOH was added, followed by incubation with agitation on an orbital shaker for 3 h at 4°C and centrifugation at 20,000 × *g* for 20 min at 4°C (Liu and Fang, 2002). The supernatant, containing the isolated ECM, was filtered through a 0.2 μm pore size membrane (Whatman, UK).

#### 2.2.8 Isolation with a Dowex cation exchange resin (named as method G)

First, the extraction buffer and Dowex cation exchange resin were prepared. The extraction buffer contained 17.8 mg of Na_2_HPO_4_ · 2H_2_O (Serva 30200.01), 27.5 mg of NaH_2_PO_4_ · H_2_O (Serva 30186), 26 mg of NaCl (KEFO 7647-14-5), 3.7 mg of KCl (Serva 26868.02), and 50 ml of distilled water. The Dowex cation exchanger was prepared by adding 1 g of Dowex cation exchange resin (Supelco 44514, Merck, Germany) to 10 ml of extraction buffer, mixing well with an automatic pipette, and incubating for 15 min. The extraction buffer was then removed using an automated pipette, and the washing procedure repeated. The extraction buffer was then removed again using an automated pipette, and 10 ml of extraction buffer was added. The prepared cation exchanger was stored at 4°C until use.

The pellet was resuspended in 1 ml of PBS by vortexing, and the suspension was divided into two 2 ml microcentrifuge tubes, to each of which 1 ml of Dowex cation exchanger was added. This was followed by incubation with agitation on an orbital shaker for 3 h at 4°C and centrifugation at 12,000 × *g* for 10 min at 4°C (Frølund et al., 1996). The supernatant, containing the isolated ECM, was filtered through a 0.2 μm pore size membrane (Whatman, UK).

#### 2.2.9 Isolation with ether solution (named as method H)

First, 10 ml of 30 mM ether solution was prepared from 112 mg of dicyclohexano-18-crown-6 (Sigma-Aldrich 158402, Merck, Germany) and 50 mM Tris–HCl, pH 8 (Tris, Serva 37180.04; HCl, VWR Chemicals BDH 20252.290).

The pellet was resuspended in 1 ml of PBS by vortexing, and the suspension was divided into two 2 ml microcentrifuge tubes, to each of which 500 μl of 30 mM ether solution was added. This was followed by incubation with agitation on an orbital shaker for 3 h at 4°C and then centrifugation at 16,000 × *g* for 20 min at 4°C (Jachlewski et al., 2015). The supernatant, containing the isolated ECM, was filtered through a 0.2 μm pore size membrane.

### 2.3 Preparation of total cell lysate (named as method CL)

Before cell lysate preparation, empty 15 ml centrifuge tubes were weighed. Next, *C. jejuni* biomass was scraped off eight plates with a sterile disposable cotton swab and added to tubes containing 4 ml of cell lysate buffer (2 mM EDTA and 1% Triton X-100 (9036-19-5, Merck) in PBS). This was followed by centrifugation at 4,400 × *g* for 20 min at 4°C. The supernatant was then removed, and the remaining pellet was weighed to calculate the amount of biomass scraped from the plates. The pellet was then resuspended in 4 ml of cell lysate buffer. This was followed by sonication with the Hielscher UP200St (Ultrasound Technology, Germany) sonicator (cycle, 90%; amplitude, 90; power 200 W) for four rounds of 5 min each. This was followed by centrifugation at 18,000 × *g* for 5 min at 4°C. Cell lysates were filtered through a 0.2 μm pore size membrane and stored at −20°C until further use.

### 2.4 Analysis of isolated ECM

The isolated ECM samples were analyzed for polysaccharides, proteins and eDNA. The polysaccharides were analyzed using the phenol-sulfuric acid method. In addition, SDS-PAGE and periodic acid-Schiff staining were used to detect glycoproteins and polysaccharides and SDS-PAGE and Alcian blue staining were used to detect acidic polysaccharides and polysaccharides with sulfate groups. The total protein content of the samples was determined using the commercial colorimetric DC protein assay (Bio-Rad, USA). The proteins in the sample were also analyzed by SDS-PAGE and Coomassie staining. The eDNA content was determined by agarose gel electrophoresis.

#### 2.4.1 Phenol-sulfuric acid method

The phenol-sulphuric acid method is a quantitative spectrophotometric method for determining the concentration of carbohydrates in a sample. A calibration curve was established with glucose standard solutions prepared from a stock concentration of 1 mg/ml. A glucose solution of the indicated concentration was prepared in sterile glass tubes to a final volume of 100 μl in two technical replicates of each concentration. Samples were prepared in two technical replicates by adding 50 μl of dH_2_O to 50 μl of the sample. 50 μl of 80% phenol (Sigma-Aldrich P9346) was then added to all tubes and the contents shaken. Then 2 ml of sulfuric acid (Carlo Erba Reagents 410301, Italy) was added to each tube and incubated for 10 min at room temperature. After 10 min, 1 ml of each solution was transferred to cuvettes and the absorbance was measured at 490 nm using a Lambda-25 spectrophotometer (PerkinElmer, USA). The concentrations of polysaccharides in each ECM sample were then determined using the calibration curve. For the statistical analysis of the results, an ordinary one-way ANOVA with Dunnett’s comparison tests was performed in GraphPad Prism (GraphPad Software, San Diego, United States).

#### 2.4.2 DC protein assay kit analysis

The commercial DC Protein Assay Kit (Bio-Rad, USA) was used to determine the protein concentration in ECM samples. A calibration curve for bovine serum albumin was established to determine the protein concentration. The calibration curve for bovine serum albumin (Sigma-Aldrich 9048-46-8) was generated with a stock concentration of 10 mg/ml. 5 μl of the standard solution of bovine serum albumin or 5 μl of each ECM sample was applied to a microtiter plate. To ensure comparability with the calibration curve, 2- and 5-fold dilutions were also prepared for the samples. Subsequently, 25 μl of reagent A’ (Bio-Rad, USA) and 200 μl of reagent B (Bio-Rad, USA) were added to all wells of the microtiter plate and incubated on an orbital shaker for 5 s while shaking. This was followed by a 15-min incubation at room temperature. After 15 min, the absorbance was measured at 750 nm using a Tecan Infinite M1000 spectrophotometer (Tecan, Switzerland). The protein concentration of each sample was determined using the calibration curve. For the statistical analysis of the results, an ordinary one-way ANOVA with Dunnett’s comparison tests was performed in GraphPad Prism (GraphPad Software, San Diego, United States).

#### 2.4.3 SDS-PAGE and different staining methods

The protein content of the ECM samples was analyzed by SDS-PAGE. 30 μl of ECM was mixed with 5 μl of 6× loading buffer and loaded onto a 1.5 mm 10% polyacrylamide geL. The Amersham Low Molecular Weight Calibration Kit for SDS electrophoresis (Cytiva 17-0446-01, GE HealthCare, USA) was used as a size marker. Electrophoresis was performed in 1X SDS buffer in a SDS-PAGE device (Mini Protean II, Bio-Rad, USA). A constant current of 35 mA/gel was used for protein separation.

##### 2.4.3.1 Glycoprotein and polysaccharide detection

Periodic acid staining and Schiff’s reagent (Sigma-Aldrich, USA) were used to detect glycoproteins and polysaccharides following manufacturer’s instructions. The SDS-PAGE gel was first fixed in 50% methanol (Carlo Erba Reagents 412721) for 30 min. The gel was then washed twice with 3% glacial acetic acid (J.T.Baker 64–19-7) solution, each wash lasting 20 min. This was followed by incubation with oxidation reagent (to prepare 100 ml of oxidation reagent, 1 g of periodic acid (Sigma-Aldrich P7875) was weighed, 3 ml of 100% glacial acetic acid and up to 100 ml of dH_2_O were added) for 15 min with constant stirring. The gel was then washed three times with 3% glacial acetic acid solution, each wash lasting 15 min. The gel was then incubated with Schiff’s reagent (Sigma-Aldrich S5133) for 35 min and then with reducing reagent (0.5 g Na₂S₂O₅ (Sigma-Aldrich 71932, Merck) was used to prepare 100 ml reducing reagent and added up to 100 ml dH_2_O) for 5 min. It was washed with 3% glacial acetic acid solution for 5 min and finally with ultrapure water for 5 min. The gel was photographed with the camera of a cell phone.

##### 2.4.3.2 Polysaccharide detection

To detect acidic polysaccharides and polysaccharides with sulphate groups, staining with the dye Alcian blue was performed. The SDS-PAGE gel was first fixed in EAW (Ethanol, Acetic acid and distilled Water) solution for 4 h (to prepare 100 ml of EAW, 40 ml of absolute ethanol, 5 ml of 100% glacial acetic acid and up to 100 ml of dH_2_O were added). The gel was then incubated in Alcian blue solution for 30 min (to prepare 100 ml of Alcian blue solution, 0.5 g of Alcian blue dye (Merck 1.05234.0010) was weighed out, 2 ml of 100% glacial acetic acid and up to 100 ml of dH_2_O were added). After incubation, the gel was washed twice with 2% glacial acetic acid solution, each wash lasting 15 min, and then incubated overnight. The gel was photographed the next day using a ChemiDoc gel documenter (Bio-Rad, USA) and a cell phone camera.

##### 2.4.3.3 Protein detection

The proteins on the SDS-PAGE gels were detected using Coomassie Brilliant Blue staining. After electrophoresis, the gel was transferred to a Petri dish containing Coomassie dye (ThermoScientific, USA). The gel was incubated in the solution for 1 h on an orbital shaker at room temperature. After 1 h of incubation, destaining was performed with 30% destaining solution (300 ml ethanol (Carlo Erba Reagents 4146072), 100 ml glacial acetic acid, and 600 ml dH_2_O were used to prepare 1 L solution), which was changed three times every 15 min. A 10% destaining solution (140 ml ethanol, 50 ml glacial acetic acid and 810 ml dH_2_O) was then added and the gel was incubated overnight. The gel was photographed the next day using a ChemiDoc gel documentation device (Bio-Rad, USA).

#### 2.4.4 Agarose gel electrophoresis

Agarose gel electrophoresis was used to analyze the DNA content of the ECM. A 1% agarose gel was prepared. The gel was prepared by weighing 0.5 g of agarose (Sigma-Aldrich A9539) into a flask and dissolving it in 50 ml of TAE (40 mM Tris-acetate, pH 8, 1 mM EDTA) electrophoresis buffer. The melted agarose was cooled to 60°C and 5 μl of the intercalating dye SYBR Safe (Invitrogen S33102) was added. The solution was poured into an electrophoresis beaker (Bio-Rad, USA). After the gel had set, 1X TAE buffer was added and 20 μl of the sample was applied to the pockets to which 4 μl of 6-fold loading buffer had already been added. The DNA size marker 1 kb GeneRuler (Thermo Scientific, USA) for the determination of fragment size above 1 kbp (6 μl) was also applied to the gel to which 1 μl of 6-fold loading buffer had been added. Agarose gel electrophoresis was performed at a constant voltage of 80 V for approximately 40 min. After completion of electrophoresis, the gel was transferred from the beaker to a UV beam gel imager (UVItec, UK) or an Image Lab Touch gel imager (Bio-Rad, USA).

## 3 Results and discussion

After extraction, we determined the eDNA content in each sample, the protein content and the polysaccharide content to evaluate the yield of these macromolecules for each protocol. We used eight different protocols that utilized NaCl, centrifugation, heating in Na_2_CO_3_, EDTA, NaOH, formaldehyde and NaOH, the cation exchanger Dowex, and an ether solution. Different protocols for isolating the ECM of *C. jejuni* yielded variable amounts of polysaccharides, proteins, and eDNA. Interestingly, Flemming and Wingender (2010) and Aguilera et al. (2008) came to similar conclusions for ECM of other types of microbial biofilms. Numerous studies have endeavoured to develop a protocol for the optimal isolation of ECM components (Liang et al., 1992; Tabouret et al., 1992; Tapia et al., 2009; Wu and Xi, 2009), however, a universal method for ECM isolation remains elusive and challenging in terms of cost-effectiveness, simplicity, and applicability to different types of components and bacteria (Aguilera et al., 2008; Chiba et al., 2015).

To evaluate the isolated ECM samples, we assessed which isolation methods are most suitable for (1) isolating the ECM of *C. jejuni*, (2) isolating individual major components of the ECM, and (3) yielding the highest concentrations of individual ECM components. Table 3 summarizes the main components of the ECM in samples prepared by different isolation methods in our study.

**Table 3:** Analysis results for determining the presence of major extracellular matrix components in samples prepared using different methods for isolating the extracellular matrix of *C. jejuni*, including the estimation of total isolation time and assessment of isolation performance.

Method	Label	Agarose gel electrophoresis	Protein concentration	Coomassie staining	Polysaccharide concentration	PAS staining	Alcian blue staining	Estimation of total isolation time	Assessment of isolation performance
Isolation with sodium chloride	A	+	-	+	+	+	+	35 min	+++
Isolation by centrifugation	B	++	+	++	++	+++	++	45 min	+++
Supernatant	SV	++	+	++	+	+++	++	18 min	+++
Isolation by heating in sodium carbonate	C	++	+	+++	+	+++	+++	85 min	++
Isolation with EDTA	D	+	-	++	+	+	-	225 min	++
Isolation with sodium hydroxide	E	++	++	++	++	+	+	225 min	++
Isolation with formaldehyde and sodium hydroxide	F	+	+	+	+	+	+	285 min	++
Isolation with cationic Dowex exchanger	G	+	+	+++	+	+++	+	215 min	+
Isolation with ether solution	H	+++	-	+	+	+	+	225 min	++
Cell lysate	CL	++	+	++	+	+++	++	80 min	+

Protein content is indicated as very high (+++; > 20 mg/ml), high (++; 10–20 mg/ml), low (+; 0.1–10 mg/ml), and very low (−; < 0.1 mg/ml). Polysaccharide content is indicated as very high (+++; > 0.5 mg/ml), high (++; 0.25–0.5 mg/ml), low (+; 0.1–0.25 mg/ml), and very low (−; < 0.1 mg/ml). The table also includes an estimate of the time required for isolation without reagent preparation and an evaluation of the performance of each isolation method. The ease of the methods is labelled as follows: easy to perform (+++), moderately difficult (++), and difficult (+).

### 3.1 Polysaccharides

**Figure 1:**
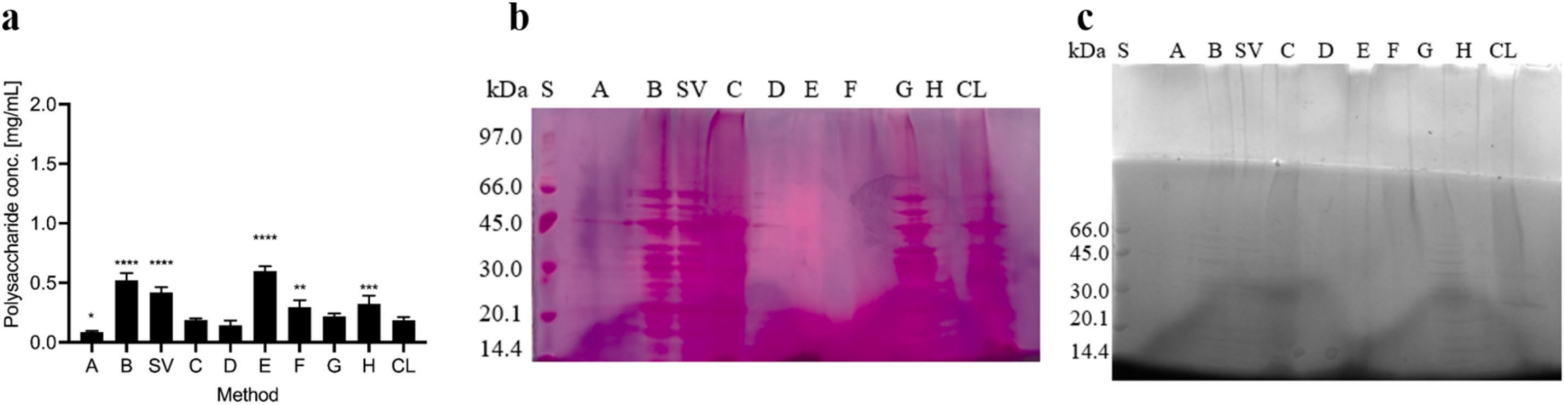
Polysaccharides in the ECM isolates of *Campylobacter jejuni*. **(A)** Polysaccharide content as revealed by the phenol-sulphuric acid method. The mean values and standard deviations of two measurements are shown. Dunnett’s multiple comparisons with the control cell lysate tested at significance levels of * *p* < 0.05, ** *p* < 0.01, *** *p* < 0.001, and **** *p* < 0.0001. The ANOVA results were significant [*F* (9, 3) = 60.98, *p* < 0.0001]. **(B)** SDS-PAGE analysis with periodic acid-Schiff staining. **(C)** SDS-PAGE analysis with Alcian blue staining. Samples were obtained by sodium chloride isolation (A), centrifugation (B), a procedure that yielded a supernatant of weakly bound extracellular matrix components (SV), heating in sodium carbonate (C), EDTA (D), sodium hydroxide (E), formaldehyde and sodium hydroxide (F), Dowex cation exchanger (G) and ether solution (H). Total cell lysate (CL) was also tested.

The polysaccharide content in samples obtained according to different extraction protocols was analyzed using the phenol-sulfuric acid method and SDS-PAGE followed by periodic acid-Schiff or Alcian blue staining. First, polysaccharide concentrations were determined using the phenol-sulfuric acid method (Figure 1A). The highest polysaccharide concentrations were found in isolates obtained with both centrifugation methods (B and SV) and with NaOH (E). In other ECM isolates and in the cell lysate (CL), lower but comparable polysaccharide concentrations were found. The lowest polysaccharide concentrations were found in isolates obtained with NaCl (A), EDTA (D), and by heating in Na_2_CO_3_ (C). Despite these differences, all the methods yielded polysaccharide concentrations of less than 0.5 mg/ml. Our results indicate that the methods that most effectively isolate high polysaccharide concentrations from the ECM of *C. jejuni* employ either NaOH (E) or centrifugation only (B and SV).

**Figure 2:**
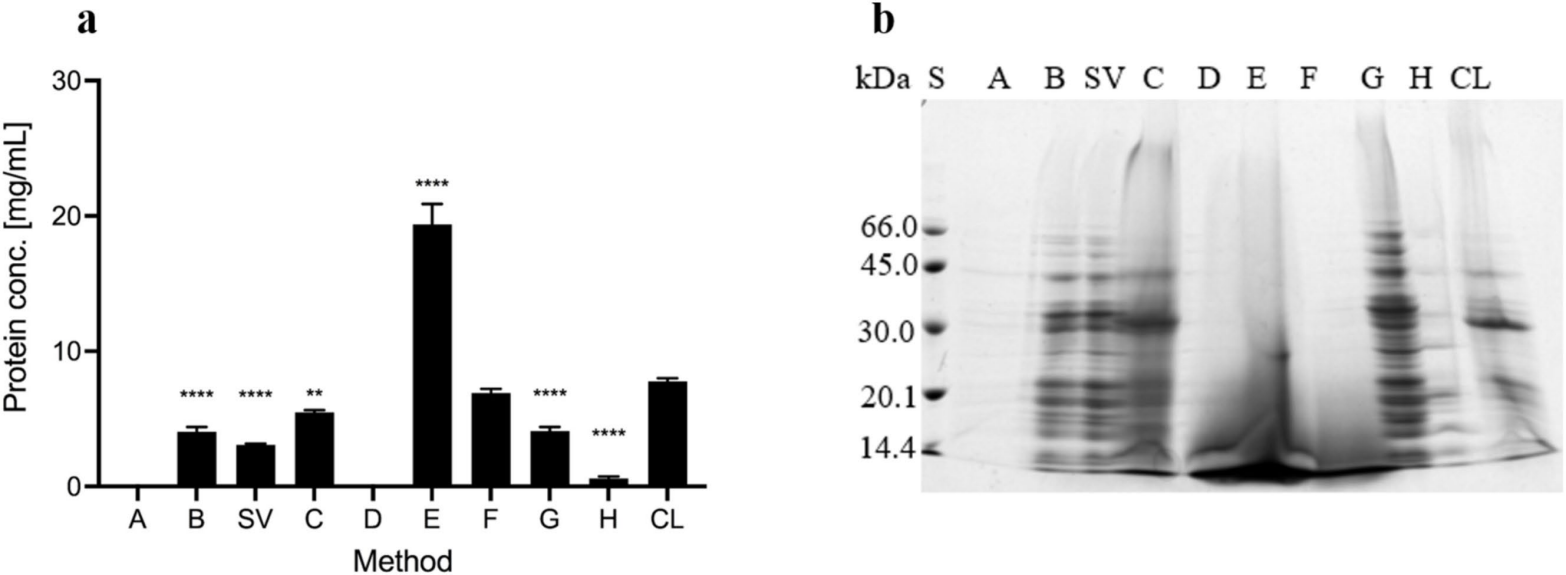
Proteins in the ECM isolates of *Campylobacter jejuni*. **(A)** Protein content as revealed by the DC Protein Assay. The mean values and standard deviations of three measurements are shown. Dunnett’s multiple comparisons with the control cell lysate tested at significance levels of p** < 0.01 and ****p < 0.0001. The ANOVA results were significant [*F* (7, 16) = 286.1, *p* < 0.0001]. **(B)** SDS-PAGE analysis with Coomassie Brilliant Blue staining. Samples were obtained by sodium chloride isolation (A), centrifugation (B), a procedure that yielded a supernatant of weakly bound extracellular matrix components (SV), heating in sodium carbonate (C), EDTA (D), sodium hydroxide (E), formaldehyde and sodium hydroxide (F), Dowex cation exchanger (G) and ether solution (H). Total cell lysate (CL) was also tested.

SDS-PAGE and periodic acid-Schiff staining (Figure 1B) showed that many individual bands were present in the isolates obtained by centrifugation (B and SV), heating in Na_2_CO_3_ (C), and the cation exchanger Dowex (G), and in the cell lysate. The patterns of these bands resemble those of SDS-PAGE and Coomassie blue staining (Figure 2B), suggestive of glycoproteins. In other isolates, no glycoproteins or polysaccharides were isolated, or the stains were less distinct.

SDS-PAGE and Alcian blue staining (Figure 1C) revealed similar patterns of acidic polysaccharides or polysaccharides with sulfate groups as SDS-PAGE and Coomassie blue staining (Figure 2B), indicating the presence of glycoproteins. No or very few glycoproteins were isolated with NaCl (A), EDTA (D), and ether solution (H). The presence of high-molecular-weight acidic polysaccharides or polysaccharides with sulfate groups was also detected by the methods using centrifugation (B and SV), heating in Na_2_CO_3_ (C), formaldehyde and NaOH (F), and the cation exchanger (G).

### 3.2 Proteins

The protein concentration was measured with the DC Protein Assay kit (Figure 2A). No protein was found in the isolates obtained with NaCl (A) and EDTA (D). The highest protein concentration was detected in the isolate obtained with NaOH (E), which is probably due to protein precipitation by denaturation with NaOH. Lower and comparable protein concentrations were detected in all other isolates and the cell lysate (CL). Protein concentrations were below 10 mg/ml in all samples except the sample obtained with NaOH, in which the protein concentration was 19 mg/ml ± 1.5 mg/ml. As such, this method is the most effective for isolating the highest protein concentrations from the ECM of *C. jejuni*, however, these proteins are most likely denatured and have lost their functionality.

SDS-PAGE and Coomassie staining (Figure 2B) detected proteins in all the isolates. Based on the intensity of the stains, a significant amount of proteins was detected in the cell lysates and all isolates, except isolates obtained with NaCl (A) and ether solution (H), in which fewer proteins were present. The same protein patterns were determined in isolates obtained with both centrifugation methods (B and SV), the cation exchanger Dowex (G), NaCl (A), and ether solution (H). The protein patterns of these samples differed from those of the cell lysate (CL). The protein pattern of the isolate obtained by heating in Na_2_CO_3_ (C) was similar to the protein pattern of the cell lysate. In other isolates (D, E, and F), predominantly low-molecular-weight, possibly denatured proteins (< 30 kDa) were detected (Figure 2B).

### 3.3 eDNA

**Figure 3:**
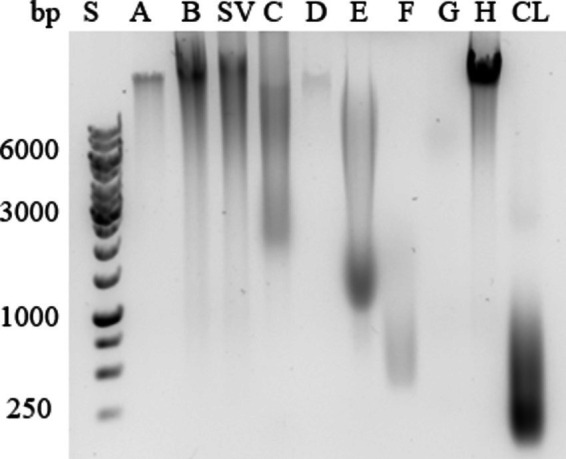
Extracellular DNA in the ECM isolates of *Campylobacter jejuni*. A representative example of agarose gel electrophoresis. Samples were obtained by sodium chloride isolation (A), centrifugation (B), a procedure that yielded a supernatant of weakly bound extracellular matrix components (SV), heating in sodium carbonate (C), EDTA (D), sodium hydroxide (E), formaldehyde and sodium hydroxide (F), Dowex cation exchanger (G) and ether solution (H). Total cell lysate (CL) was also tested.

Agarose gel analysis (Figure 3) revealed the presence of high-molecular-weight eDNA in isolates obtained with NaCl (A), both centrifugation methods (B and SV), EDTA (D), and ether solution (H), the latter showing the strongest signal. The isolate obtained by heating in Na_2_CO_3_ (C) contained fragments of >10 kbp and < 2 kbp. The isolate obtained with NaOH (E) contained fragments of 1–10 kbp. The isolate obtained with formaldehyde and NaOH (F) contained fragments of 0.5–2 kbp. The isolate obtained with the cation exchanger Dowex (G) contained a weak band of a fragment of >6 kbp. Cell lysates contained fragments of <1 kbp.

Mostly high-molecular-weight eDNA was present in the ECM of *C. jejuni*, and only cell lysates contained low-molecular-weight eDNA. This is expected because the cell lysates were sonicated during preparation, which fragmented the DNA. Our findings indicate that the most suitable method for isolating high concentrations of high-molecular-weight DNA is extraction with ether solution (H; Figure 3).

### 3.4 ECM

An overview of the methods previously used for investigating ECM, divided into extraction and purification techniques, biofilm quantification and ECM analysis (Table 2) shows the variety of methods used for ECM studies. For accurate analysis of the ECM in *C. jejuni* biofilms, the ECM must first be extracted and purified, which was usually achieved by ethanol or acetone precipitation in combination with centrifugation (Jowiya et al., 2015; Yu et al., 2020), which favours the isolation of polysaccharides and proteins. Each method can illuminate a part of the ECM mosaic from a different angle, so that one can only interpret the results correctly if one knows the method well. In *ex situ* research, sample isolation and extraction is the first step, and it is extremely important to know and understand which part of the mosaic we can investigate with it. Here we have provided an overview of how the different isolation or extraction protocols can lead to different ratios and qualities of ECM components. The selection of the extraction method is an important step that can influence the outcome of the study. However, when selecting the method, not only the quantity of isolated macromolecules should be considered, but also their quality, as some extraction methods that yield the largest quantity are also destructive (e.g., NaOH for proteins). Therefore, when selecting the method for ECM extraction, the intended downstream analysis should be considered, e.g., preference of proteins for proteomics and polysaccharides for glycomics, but also the impact of the chemicals used in the purification as they may affect the quality of samples. Finally, it is recommended to use more than one method for ECM isolation in a study to compensate for any bias in ECM composition due to the isolation method.

## 4 Conclusion

Different isolation and extraction protocols for the extracellular matrix enriched different molecular components, resulting in very different ECM samples. For the isolation of *C. jejuni* ECM and its major components, the centrifugation method, the method in which the supernatant is obtained from weakly bound components, and heating in Na_2_CO_3_ were found to be the most suitable methods. These methods isolated all biopolymers and are simple, reliable and fast. The isolation methods using NaCl and EDTA were less suitable because they isolated lower amounts of eDNA and polysaccharides and failed to isolate proteins. All other isolation methods were considered suitable. The isolation method using NaOH isolated proteins and polysaccharides at the highest concentrations most effectively, but they were degraded. The ether dissolution method was best suited for the isolation of eDNA because it isolates high-molecular-weight DNA. Depending on the intended subsequent use or analytical method for the ECM sample, the isolation protocol should be carefully selected, ideally using more than one protocol to obtain more meaningful conclusions, as different protocols result in different compositions of the main ECM components.

## Data Availability

The raw data supporting the conclusions of this article will be made available by the authors, without undue reservation.
